# Optimizing SureQuant for Targeted Peptide Quantification: a Technical Comparison with PRM and SWATH-MS Methods

**DOI:** 10.1021/acs.analchem.4c03622

**Published:** 2024-10-28

**Authors:** Minia Antelo-Varela, Dirk Bumann, Alexander Schmidt

**Affiliations:** Biozentrum, University of Basel, Spitalstrasse 41, Basel CH-4056, Switzerland

## Abstract

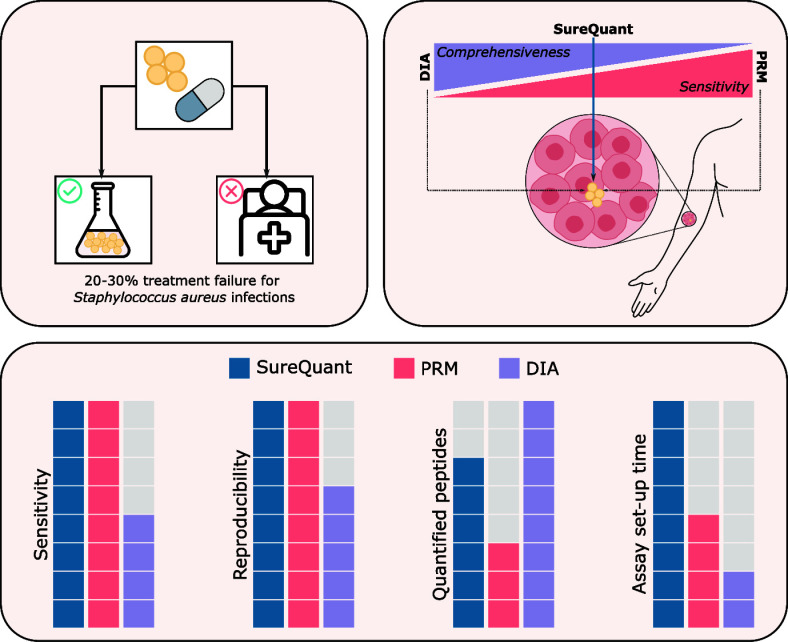

Bacterial infections are a major threat to human health worldwide. A better understanding of the properties and physiology of bacterial pathogens in human tissues is required to develop urgently needed novel control strategies. Mass spectrometry-based proteomics could yield such data, but identifying and quantifying scarce bacterial proteins against a preponderance of human proteins is challenging. Here, we explored the recently introduced SureQuant method for highly sensitive targeted mass spectrometry. Using a major human pathogen, the Gram-positive bacteria *Staphylococcus aureus*, as an example, we evaluated several parameters, including the number of targets and intensity thresholds, for optimal qualitative and quantitative protein analysis. By comparison, we found that SureQuant achieved the same quantitative performance as standard parallel reaction monitoring while allowing accurate and precise quantification of up to 400 targets. SureQuant also surpassed the sensitivity and quantification capabilities of global data-independent acquisition methods. Finally, to facilitate method development, we provide optimized MS parameters for the sensitive quantification of different peptide panel sizes. This study provides a foundation for the broader application of SureQuant in the analysis of clinical specimens containing trace amounts of bacterial proteins as well as other studies requiring ultrasensitive detection of low-abundant proteins.

## Introduction

Bacterial infections have become one of the largest threats for public health of this century.^1^ The increase in antimicrobial resistance, along with a stagnation in the development of new antibiotics, has precipitated a surge in cases of infections that are no longer responsive to current antibiotic therapies. In addition, some infections respond poorly to antimicrobial treatment even if the causative strain appears susceptible in laboratory tests. This is particularly critical in the case of *Staphylococcus aureus*, a Gram-positive bacterium that causes a wide variety of infections associated with substantial morbidity and mortality.^2−4^ These discrepancies suggest that the laboratory conditions fail to recapitulate relevant aspects of *S. aureus* physiology in tissue microenvironments.^5,6^ A better understanding of this in-patient physiology of *S. aureus* is required to develop urgently needed novel control strategies.

Mass spectrometry (MS)-based proteomics has the potential to reveal bacterial activities and properties in patient biopsies by detecting proteins associated with their metabolism, virulence, and host interaction.^7,8^ As examples, the detection of virulence factors would reveal the activation of a virulence program, the detection of antimicrobial resistance factors could suggest decreased susceptibility to treatment, and the detection of surface proteins could suggest potential vaccine candidates. Together, such data would provide benchmarks for bacterial *in vivo* properties that can be compared to bacterial properties under various *in vitro* conditions. This could help to develop more physiologically relevant *in vitro* assays for developing novel antimicrobials and vaccines.

However, the abundance of host material relative to bacterial cells complicates the detection and quantification of bacterial proteins. This can be particularly challenging in tissues with heterogeneous pathogen distribution including regions with only sparse bacteria. In tissue infections, host-to-bacterial protein ratios can range from 10:1 to 100:1, while in cell culture infections, this ratio may vary from 75:1 to 100:1, or as low as 7.5:1 with high bacterial replication.^5,6^ Clinical proteomics has predominantly relied on the utilization of discovery proteomics techniques based on data-dependent acquisition (DDA) or data-independent acquisition (DIA) like SWATH-MS.^9^ While both these methods offer extensive coverage, they exhibit a bias toward highly abundant proteins, frequently overlooking low-abundance targets that are critical in this context. To tackle this challenge, targeted-MS techniques such as selected reaction monitoring (SRM) and parallel reaction monitoring (PRM) have emerged as promising solutions.^10−12^ These techniques boast highly sensitive, reproducible, and rapid detection capabilities, enabling accurate quantification of a predetermined panel of target peptides. However, any targeted acquisition strategy involves a compromise between the number of targeted peptides and the sensitivity and selectivity of those measurements,^13,14^ often limiting the depth of coverage per LC-MS analysis to only a few proteins. To address these challenges, trigger-based acquisition methods have been recently developed with SureQuant, allowing large-scale PRM analysis.^15−17^ Derived from the conventional IS-PRM method,^18^ SureQuant leverages isotopically labeled peptides to dynamically guide the instrument in real-time, eliminating the need for retention time scheduling thereby preserving depth of coverage and sensitivity.^19,20^ This method is particularly promising for uncovering the in-patient physiology of *S. aureus* in complex host environments. Nevertheless, there remains a gap in our understanding of both the limitations and strengths of this method as well as the impact of the various MS settings on quantitative performance.

In this study, we have systematically modified various parameters of SureQuant to optimize its performance for the sensitive detection and quantification of 326 different *S. aureus* peptides that are involved in a wide variety of functions including virulence, stress defense, metabolism, and housekeeping functions. We compared SureQuant with standard PRM and could demonstrate equivalent quantitative capabilities and sensitivity, but much higher coverage per run. Additionally, a comparison with SWATH-MS showed superior sensitivity of SureQuant for the quantification of low-abundance proteins. With its high multiplexing capabilities and sensitivity, SureQuant offers a valuable alternative for targeted proteomic analyses when the proteins of interest are only minor components of a highly complex mixture.

In summary, this research contributes to ongoing advancements in targeted proteomics and emphasizes the importance of optimizing and evaluating specific methodologies like SureQuant for clinically significant research studies. Our results pave the way for the development of innovative diagnostic and therapeutic approaches against *S. aureus* infections, and potentially other pathogens, by enabling precise quantification of low abundant targets within complex clinical samples.

## Experimental Section

All LC-MS analyses were conducted in triplicate to ensure data reproducibility. Further details on the experimental design and data analysis are provided in the Supporting Information.

### Selection of Peptides for SureQuant Analysis

We included a total of 131 proteins comprising major virulence factors, vaccine targets, resistance markers and metabolic proteins of the human pathogen *S. aureus*. The details regarding peptide selection and synthesis, as well as the list of labeled peptides and respective transitions are detailed in the Supporting Information (Material & Methods) and Table S-1.1and 1.2.

### Panel Expansion

Our research entailed the examination of a total of 326 Stable Isotope Labeled (SIL) unique *S. aureus* peptides. To enrich our peptide data set to encompass up to 1600 peptides, we leveraged previously acquired SWATH-MS data sourced from a culture of *S. aureus* ATCC 29213 (MSSA) strain cultivated in MHB medium. Detailed list of these peptides, along with their inclusion in specific panels, is provided in the Supporting Information (Table S-2). Among these, 326 peptides remained consistent across relevant panels. Additional peptides were selected from the SWATH-MS database, based on specific criteria. Specifically, tryptic peptides carrying +2 and +3 charges, ranging between 7 to 21 amino acids, were chosen. Panels were then constructed to include 1600, 800, 400, 200, 100, and 50 targets. Throughout this process, common peptides were consistently retained whenever new sets were added.

### SureQuant Acquisition

For SureQuant acquisition, we applied the template provided in Thermo Orbitrap Exploris Series 4.1, employing the default settings that include four branches for both +2 and +3 charge states of SIL lysine and arginine residues. The standard MS parameters for SureQuant acquisition were set as follows: a spray voltage of 2500 V, no sheath or auxiliary gas flow, and a heated capillary maintained at 275 °C. Full-scan mass spectra were acquired with a scan range of 375–1600 *m*/*z*, an AGC target value of 300%, maximum injection time (IT) of 50 ms, and a resolution of 120,000. Within a 5-s cycle time per MS1 scan, heavy peptides matching the *m*/*z* (within 5 ppm) and intensity threshold set to 1e6 (for expanded panel experiment), or as defined in the inclusion list (5-fold less; Table S-3) were isolated (isolation width of 0.4 *m*/*z*) and subjected to fragmentation (nCE: 27%) by HCD with a scan range of 150–1700 *m*/*z*, maximum IT of 10 ms, AGC target value of 1000%, and a resolution of 7,500. A product ion trigger filter was then applied, conducting pseudospectral matching and triggering MS/MS events exclusively for the endogenous target peptide at the defined mass offset if at least two product ions were detected from the specified list. If triggered, the subsequent MS/MS scan for the light peptide shared the same collision energy (CE), scan range, and AGC target as the heavy trigger peptide, with an increased maximum injection time and resolution (IT: 116 ms, resolution: 60,000). For the comparison with PRM, the parameters remained as described above, except for IT of heavy peptide which was increased to 22 ms and resolution of 15,000 (Table S-4.1)

### SureQuant Data Analysis

Peak area ratios (light/heavy) of endogenous light peptides and corresponding heavy SIL peptides for the two selected product ions were exported from SpectroDive (10.4.210316.47784 (Ictíneo II). Only peptides with a) elution group *q*-value <0.01 and b) a ratio two times higher than the ratio of the SIL peptide alone (which often contain measurable light contamination) were considered for quantification. Light contamination of SIL peptides were measured for each SIL peptides alone in a HEK background without *S. aureus* in an initial PRM LC-MS analysis at the beginning of each sample set. The determined contamination ratios were subtracted from the final peptide ratios to ensure accurate quantification. After median normalization, protein abundances were calculated based on the ratios determined and the amount of SIL peptides spiked into the sample. Here, for a given protein, the peptide with the highest ratio served as the reference, assuming it most accurately represents the protein concentration in the sample. The ratios of the remaining peptides for that protein were adjusted by calculating correction factors based on the ratio of each peptide relative to the most intense peptide. The median of these correction factors across samples was used to correct the ratios of the peptides. All analyses were conducted using Rstudio version 4.3.2. and the code is provided in the Supporting Information. For multisample (ANOVA) or pairwise proteomic comparisons (two-sided unpaired *t* test, log-transformed values), we applied a permutation-based FDR of 5% to correct for multiple hypothesis testing and a s0 value of 0.1 using Perseus (v1.6.14.0).^21^

## Results and Discussion

As a trigger-based method, SureQuant relies on the efficient and specific detection of the SIL peptide to trigger the targeted MS analysis of the endogenous peptide ion. This is a two-step procedure including first the detection of precursor ion in the full mass spectra (MS1 level) and second the detection of the corresponding fragment ions in the triggered low resolution tandem mass spectra (MS2 level). Since in a SureQuant analysis 100s of ion masses are provided as potential targets for triggering MS2 spectra, stringent filters have to be applied to keep time-consuming false scan events as low as possible while detecting all targets. We therefore first evaluated and optimized these initial steps.

### Optimization of Triggering Efficiency

To optimize triggering efficiency, we first assessed the minimal SIL peptide amounts required for robust identification and quantification of their corresponding unlabeled (UL) counterparts. Therefore, we spiked decreasing amounts (10, 5, 1, 0.4, 0.02, and 0 fmol on column) of 23 SIL peptides (Table S-1.1) into a HEK (Human Embryonic Kidney) cell lysate background while maintaining consistent levels of UL peptides (10 fmol on column). Throughout the manuscript, all LC-MS analyses were performed in triplicates. As shown in Figure S-1a, the minimal amount of SIL peptide on column required to identify all targets was 5 fmol. Approximately 20% of targets were missed with 1 fmol, 36% with 0.4 fmol, and 81% with 0.02 fmol on column. When comparing 5 and 10 fmol on column, we observed a slightly better precision with 10 fmol on column, as indicated by lower coefficients of variation (CVs) (Figure S-1b) and higher number of points per peak (Figure S-1c). Consequently, all experiments described in this study used 10 fmol on column of SIL peptides.

Next, we evaluated the impact of precursor ion mass tolerances on triggering selectivity. We therefore analyzed the same peptide mixture containing 23 peptides in their light (UL) and heavy (SIL) form spiked (10 fmol on column) into a HEK matrix (Table S-1.1) applying three different mass tolerances: 10, 5, and 3 ppm. Additionally, we analyzed blank samples containing only HEK background. Our findings revealed that the number of low-resolution scans remained consistent, both for samples and blanks, with a gradual decrease observed as mass tolerance decreases (Figure S-2a), indicating that precursor mass tolerance only had a minor impact on triggering specificity. Conversely, we noted a gradual increase in high-resolution scans with decreasing mass tolerance in samples with spiked peptides, while the opposite trend was observed for the blanks (Figure S-2b). Notably, the highest stringency in mass tolerance (3 ppm) led to a slight decrease in the number of identified targets (Figure S-2c). We also observed an improvement in quantitative precision with decreasing mass tolerance, with 5 and 3 ppm yielding the most favorable results due to their high number of data points per peak (Figure S-2d). While the narrowest mass tolerance enhanced specificity and accuracy, some peptides were excluded as they fell outside the tolerance window. Since the overall impact of mass tolerance on triggering specificity was rather small, and we prioritized comprehensive identification, the experiments described in this paper employed a mass tolerance setting of 5 ppm as the best trade-off between sensitivity and selectivity.

**Figure 1:**
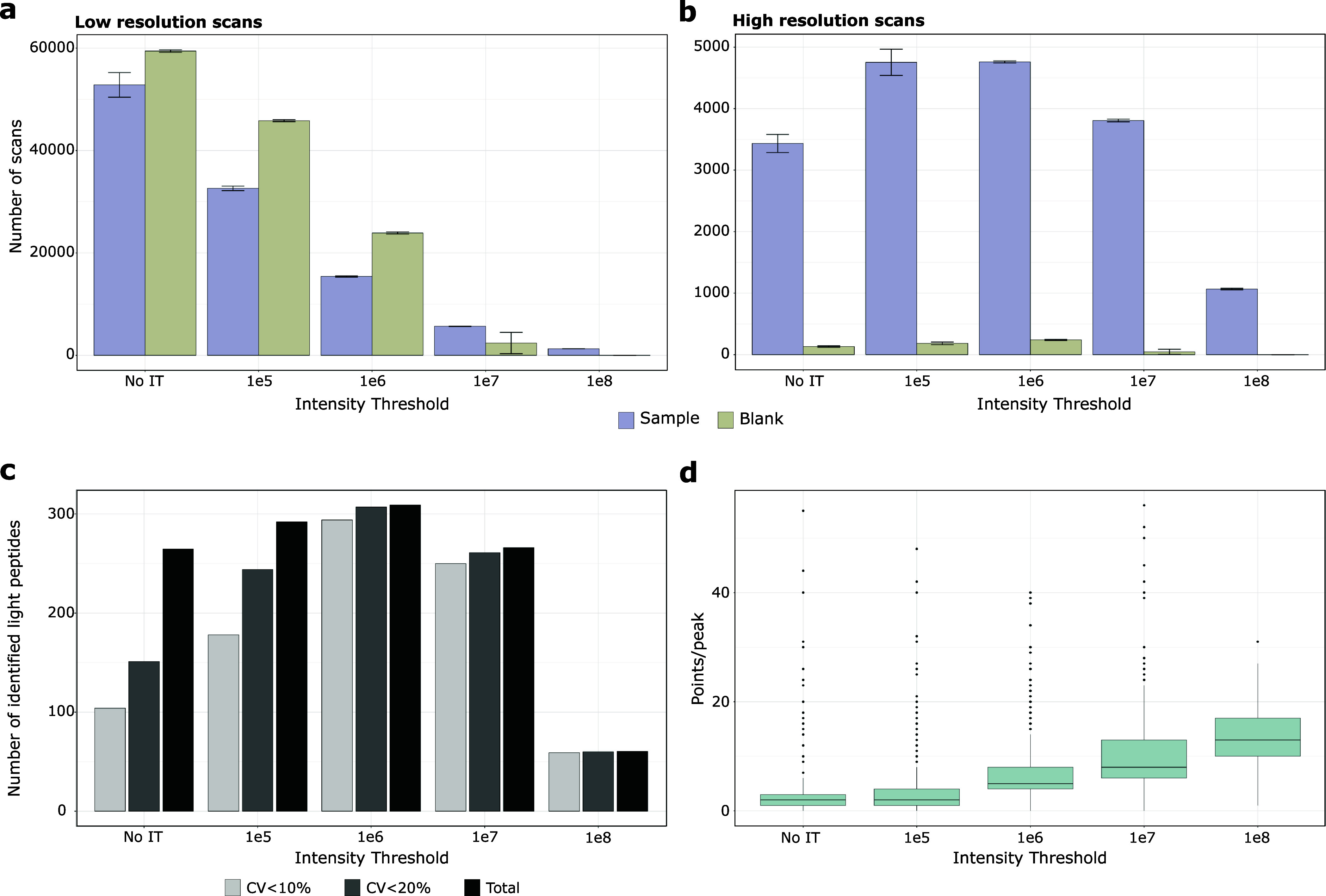
Distribution of scans based on intensity threshold for samples and blanks and impact on quantification accuracy. a) Counts of low-resolution scans. b) Counts of high-resolution scans. Results are shown with purple bars representing samples containing both light and heavy peptides in a 250 ng HEK background, and light green bars representing blank samples consisting solely of the 250 ng HEK background. The error bars display the standard deviations of the triplicate MS analyses. c) The average identifications of triplicate technical replicates and the number of peptides with CVs below defined thresholds were calculated. d) Box plot visualization of number of points per peak of all identified peptides according to the applied intensity threshold filter. The box plot displays the median (central line), the 25th and 75th percentiles (bottom and top edges of the box), and the whiskers, which extend to 1.5 times the interquartile range from the 25th and 75th percentiles. Data points beyond the whiskers are considered outliers and are shown as individual points.

In a next step, we assessed if precursor ion MS intensity thresholds could further increase triggering specificity. As shown in Figure S-2a, we noticed that even if SIL peptides were not present, corresponding low-resolution scans were still initiated. We hypothesized that applying intensity thresholds could reduce false triggering and improve specificity as already described recently.^15^ To evaluate this, we added identical amounts of SIL and UL peptides to a HEK cell lysate background and applied the following intensity threshold settings; none, 1e5, 1e6, 1e7, and 1e8. Our analysis included a defined set of 326 peptides (Table S-1) and also blank samples to determine the amount of false triggering events. As expected, the number of low-resolution scans decreased strongly with increasing intensity thresholds (Figure 1a). Interestingly, at low thresholds, even more low-resolution scans were acquired in the blank than in the spiked samples, indicating a high degree of false triggering events. Conversely, at high intensity thresholds, twice as many low-resolution scans were acquired in the spiked sample over blank, demonstrating improved target specificity. This was even more apparent from the number of high-resolution scans that showed a steady increase in specificity and reduction of scan numbers with higher thresholds (Figure 1b). Surprisingly, setting no intensity thresholds also scaled down the number of high-resolution scans. This can be primarily ascribed to the high number of false-triggered scans that diminish overall MS duty cycle and thus the acquisition of high-resolution scans. Most importantly, the observed higher specificity enabled more target identifications with higher precision (Figure 1c). While the latter increased up to the highest threshold of 1e8, the overall number of quantified targets considerably decreased, indicating that the intensity threshold was no longer met by a growing number of targets. Conversely, for no or low intensity thresholds, almost all targets were identified, but with reduced quantitative precision. As described above, this was due to the high number of false triggered low-resolution scans that limit MS duty cycle and thus acquired data points per peak (Figure 1d). With no threshold, only 2.5 points per peak could be acquired on average which was insufficient for precise quantification. Conversely, with 7 points per peak, precise target quantification was achieved at a threshold of 1e6. This is in agreement with previous findings on the minimum points required for reliable quantification required for reproducible peptide peak quantification.^22^

Naturally, the intensity of a peptide depends on both its ionization efficiency and concentration. With our 10 fmol of spiked SIL peptides, most precursor ion intensities were around 1e7 or more (Figure S3). However, not all peptide ions reached this intensity at 10 fmol and were therefore not quantified at a threshold of 1e7 (Figure 1c). To allow efficient target triggering, we propose either to adjust the concentration of each spiked SIL peptide to reach the set thresholds, or to apply individual intensity thresholds for the different targets. Notably, the concentrations of SIL peptides could not be increased much further as we started to observe light contamination peaks that promoted false identification and quantification. Moreover, for large target panels as used in this study, spiking different amounts for each peptide becomes tedious. We therefore considered the second option and applied peptide specific threshold five times below the average peak height determined from three replicates. As shown in Figure S4, this increased quantification accuracy with almost 99.5% of peptides quantified with CVs under 10%, compared to 96.7% at the 1e6 threshold, despite a 26% decrease in high-resolution scans. The improved quantitative precision is also supported by the number of average data points per peak that increased from 7 to 10. Notably, setting individual SIL peptide intensity thresholds can be done automatically by exporting intensities from the data analysis software, matching them to the transition list and importing them to the MS method template.

To conclude, our finding show that the best performance was obtained with individual, peptide specific intensity thresholds. This approach was used for all following targeted LC-MS analyses.

### Comparison of SureQuant and PRM for Targeted Peptide Quantification

**Figure 2:**
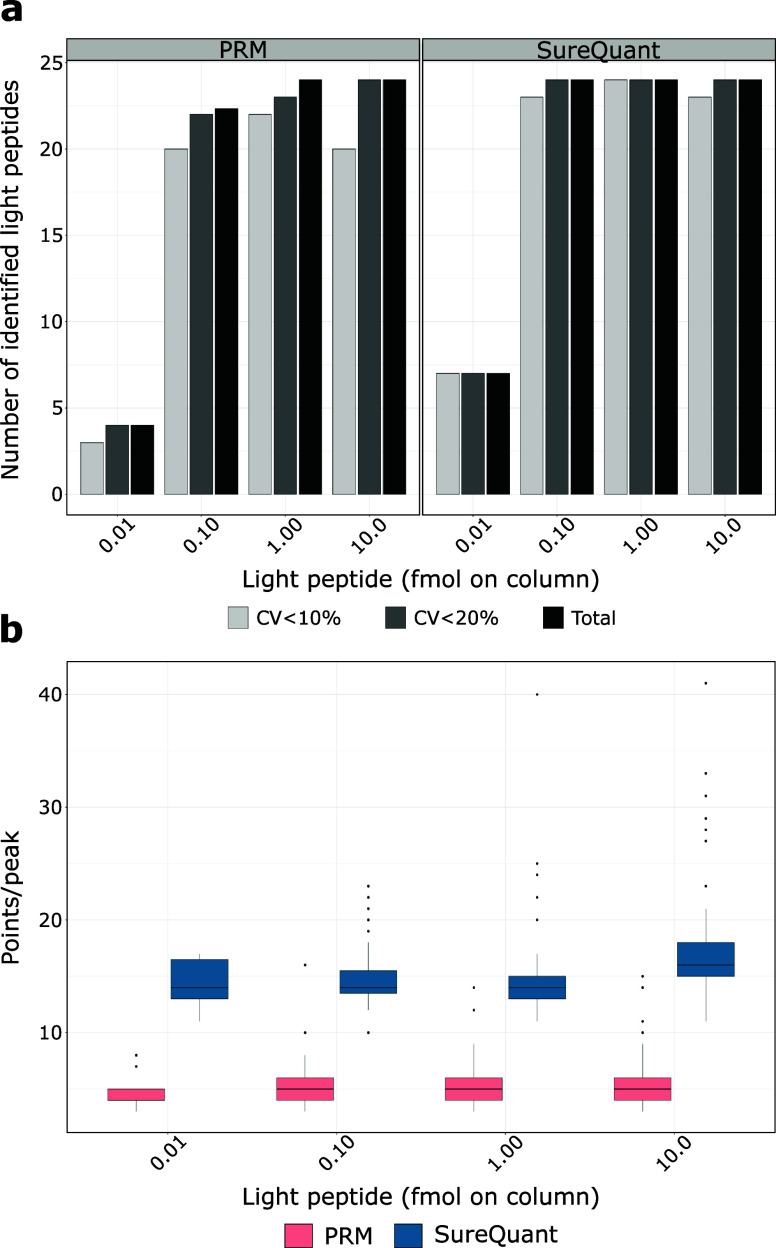
Comparative analysis of PRM and SureQuant for 24 selected peptides based on injected light peptide on column. a) The average identifications of triplicate technical replicates and the number of peptides with CVs below defined thresholds were calculated for PRM and SureQuant. b) Box plot visualization of number of points per peak of all identified peptides according to amount on light peptide injected on column. The box plot displays the median (central line), the 25th and 75th percentiles (bottom and top edges of the box), and the whiskers, which extend to 1.5 times the interquartile range from the 25th and 75th percentiles. Data points beyond the whiskers are considered outliers and are shown as individual points.

While PRM achieves sensitivities comparable to those of ELISA assays, its requirement for extended ion accumulation times and higher resolution limits its capacity to target a broad array of peptides in a single assay, often restricting the selection to a few peptides.^13^ In contrast, SureQuant addresses this limitation by employing SIL peptides to guide real-time instrument operations. However, detailed comparisons of SureQuant’s sensitivity and accuracy against established methods like PRM are still scarce. Our study aimed to directly compare these methods by analyzing 24 peptides selected from our primary set (Table S-4). To compare the quantitative performances of the two targeted methods, we prepared a dilution sample series spanning 4 orders of magnitude of UL peptides (0.01, 0.1, 1, and 10 fmol on column), maintaining a constant concentration of SIL peptides (10 fmol on column) and HEK protein background (250 ng) and analyzed them in triplicates. As shown in Figure 2a, both methods exhibit comparable and excellent peptide coverage and quantification precision, but SureQuant slightly outperformed PRM. At the lowest concentration (0.01 fmol on column), SureQuant identified nearly twice as many target peptides as PRM (Figure 2a), with all quantified peptides showing CV values below 10%. This high precision was maintained across all tested concentrations, with over 95% of targets consistently quantified with CVs under 10%. While PRM also displayed high precision, SureQuant generally presented lower CV values, for instance at 10 fmol on column, 95.8% of peptides quantified by SureQuant had CVs below 10% compared to 83% by PRM (Figure 2a). This was also reflected in the number of data points per peak with SureQuant acquiring 16 on average compared to only 6 for PRM (Figure 2b). This difference can be attributed to the operational methodologies of each system; SureQuant’s continuous monitoring of SIL peptides using fast scan speeds allowed for the immediate triggering of data collection for both SIL and endogenous peptides when intensity thresholds were exceeded, facilitating more frequent sampling and thus a higher number of data points across a peak. Conversely, cycle time in scheduled PRM is still reduced by the need for a few minutes of extra time before and after peak elution to control for retention time variations. Therefore, scheduled PRM, as performed in this study, has a lower number of data points per peak than SureQuant which likely contributed not only to higher CVs, but also to decreased identification confidence and higher *q*-values in the automated SpectroDive analysis used. Notably, this difference may vary with other PRM analysis tools, such as Skyline, or through manual data analysis.

Next, we used the data to define linear response ranges for both methods by determining the lower and upper limits of quantification (LLOQ and ULOQ) using CalibraCurve, a recently published script designed to calculate metrics of trueness and precision.^23^ This analysis included calculating the CVs for each concentration level and the average percent bias (PBav), which indicates the deviation between the true and calculated values expressed as a percentage. The performance of both methods can be exemplified using three peptides quantified across the calibration range by both methods. Here, our analysis showed that the LLOQ (∼0.01 fmol) and ULOQ were equivalent for both PRM and SureQuant (Figure S5), though SureQuant consistently demonstrated lower CVs and PBav values (Table S-5).

To conclude, our findings confirm that SureQuant slightly outperformed PRM in sensitivity and precision. It is important to note that for comparability reasons, the same MS settings were employed for both methods. Considering the number of 16 data points per peak indicates that higher resolution and fill times settings could be used for the SureQuant to further boost its sensitivity while maintaining excellent quantitative performance.^22^

### Balancing Peptide Target Numbers and MS Sensitivity

**Figure 3:**
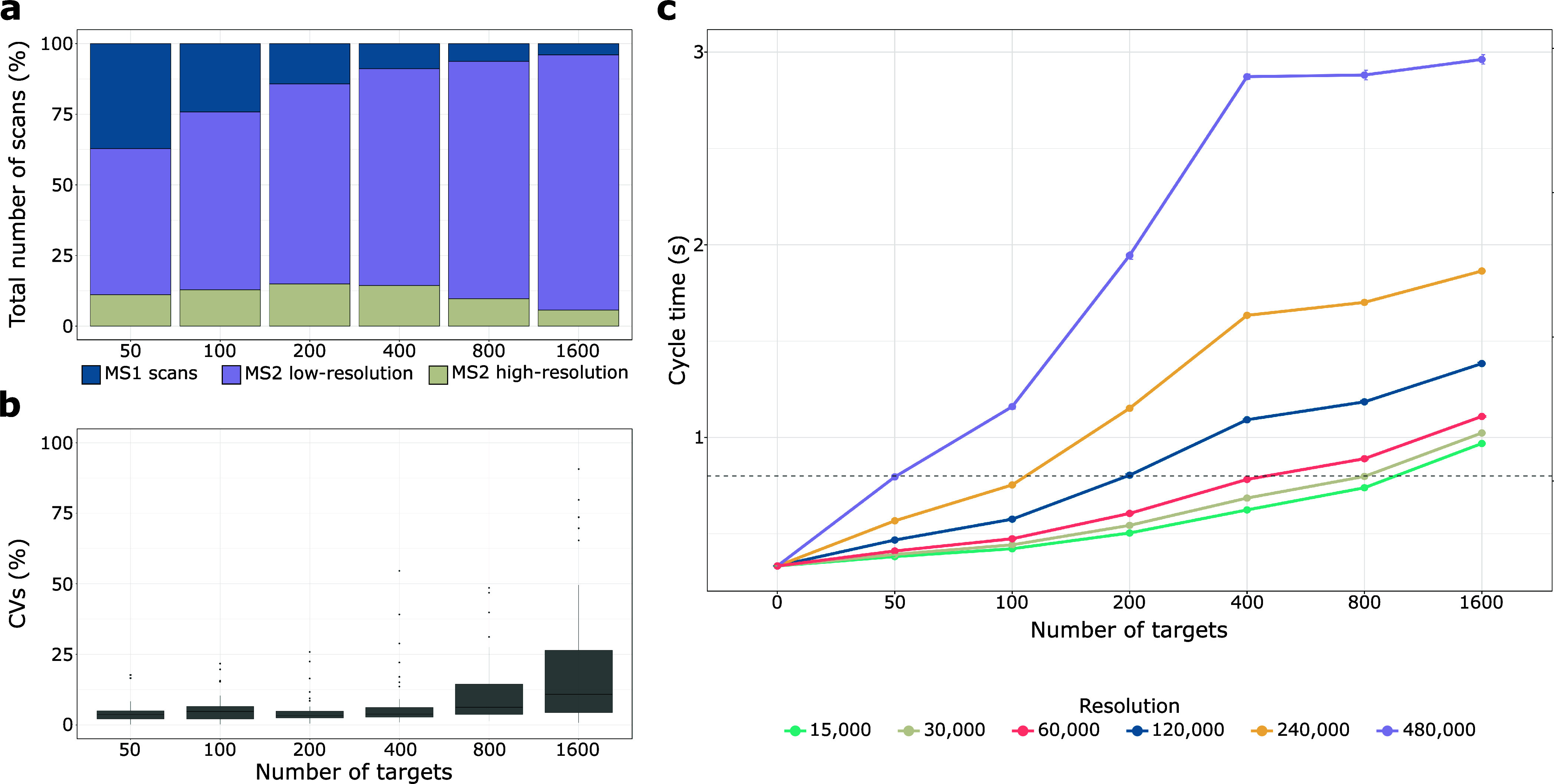
SureQuant performance as a function of the number of targets. a) Percentage of total MS1, MS2 low-resolution, and MS2 high-resolution scans normalized by the total number of scans. b) Box plot visualization illustrating the distribution of CVs for 50 consistently quantified targets across the six different conditions. The box plot displays the median (central line), the 25th and 75th percentiles (bottom and top edges of the box), and the whiskers, which extend to 1.5 times the interquartile range from the 25th and 75th percentiles. Data points beyond the whiskers are considered outliers and are shown as individual points. c) Cycle time (seconds) based on the number of targets. The resolution of 60,000 was determined from experimental data (Table S-6), while resolutions of 15,000, 30,000, 120,000, 240,000, and 480,000 were derived from theoretical calculations using experimental data. The right *y*-axis indicates the threshold at which we observed an impact of the number of targets on data quality.

The capability of PRM-based methodologies in detecting and quantifying target peptides within assays has been well-documented, typically ranging from 10 to 100 peptides per assay.^24^ Contrary to these methods reliant on elution time scheduling, SureQuant demonstrated targeting a much larger number of peptides within one LC-MS analysis, as evidenced by a recent study analyzing 340 tyrosine-phosphorylated peptides across 31 colorectal cancer tumors.^15^ Despite these impressive advancements with SureQuant, the relationship between target number, MS settings and quantitative performance as well as the upper limit of manageable peptide targets remain insufficiently understood. Here, we aimed to address this gap by incrementally increasing the number of targeted peptides from 50 up to 1600 peptides. Selection of 1274 nonpanel peptides was guided by empirical data from prior SWATH-MS analysis. Throughout our study, the inclusion of 326 SIL peptides remained consistent when relevant based on panel size, with a set of 50 consistently quantified peptides serving as benchmarks for data quality assessment. Additional information on panel construction is available in the Supporting Information (Table S-2). We evaluated MS instrument time allocation by computing the number of MS1, low-resolution MS2, and high-resolution MS2 scans, excluding scans during column equilibration. As shown in Figure 3a, the largest portion of scan time was dedicated to low-resolution scans, increasing alongside targeted peptide numbers. Conversely, MS1 scans decreased with rising target numbers, while high-resolution MS2 scans peaked at 400 targets before declining (Figure 3a). Since the number of SIL peptides remained consistent at 326, the decline of high-resolution scans in combination with the increase of low-resolution scan for the two largest panels indicated that MS scan time was becoming limiting. This was also reflected in a strong increase in CVs for 800 and 1600 targets determined from the same 50 peptides quantified across all samples (Figure 3b). Therefore, we identified 400 targets as the threshold before quantitative performance was considerably decreasing.

In every targeted MS analysis, there is a trade-off between target number and MS scan time (MS sensitivity). For all SureQuant MS analysis described so far, we employed the proposed MS settings provided in the vendor’s template (resolution of 7500 for low resolution; 60 000 for high-resolution scans). Considering the high number of low-resolution scans for large target numbers and the good quantitative performance achieved, we propose to keep the scan rate for the low-resolution at maximal speed to free valuable MS scan time for the high-resolution scans of the actual target peptides. Therefore, we next employed the scan numbers and times determined for the different target numbers to predict expected MS cycle times for different high-resolution settings and target numbers (Table S-6). Based on our previous results, we calculated the maximal MS cycle time (0.8s) that still resulted in 7 data points per peak to enable precise peptide quantification (for the LC setup -60 min active gradient) used in this study). This allowed us to predict optimal MS resolution settings for different target numbers (Figure 3c). For instance, 800 targets can still be precisely quantified using a resolution of 30 000, while for up to 50 targets, resolution can be increased to 480 000 without expecting much loss on quantitative performance. Apparently, the precise quantification of 1600 peptide targets is challenging, since even at lowest resolution, cycle times remain above the threshold. Notably, we did not use a resolution of 7 500, but considering the low reduction of cycle times from 60 000 to 15 000, we would not expect to reach 0.8s cycle time with the lowest MS resolution settings. If such large target numbers need to be analyzed, multiple LC-MS analyses with smaller, shared target lists or scheduled elution time windows^25,26^ should be used. It is also worth mentioning that these calculations assume an equal distribution of eluting peptide ions across the LC gradient. For high/low populated elution time periods lower/higher MS resolution settings need to be considered to maintain good quantitative performance. Recent advancements in mass analyzers, such as the Stellar, may shift these numbers by offering improved scan speeds and multiplexing capabilities, which could help mitigate some of the limitations observed in our simulations. Although the fundamental trade-offs between MS sensitivity and target number remain, new technologies may allow for larger target lists without compromising quantitative performance.

To conclude, we demonstrate a trade-off between MS sensitivity and target number for precise quantification with SureQuant. These findings underscore the importance of optimizing target list size and MS settings.

### Enhanced Sensitivity of SureQuant Revealed in Low Abundance Target Analysis: a Comparative Study with SWATH-MS

**Figure 4:**
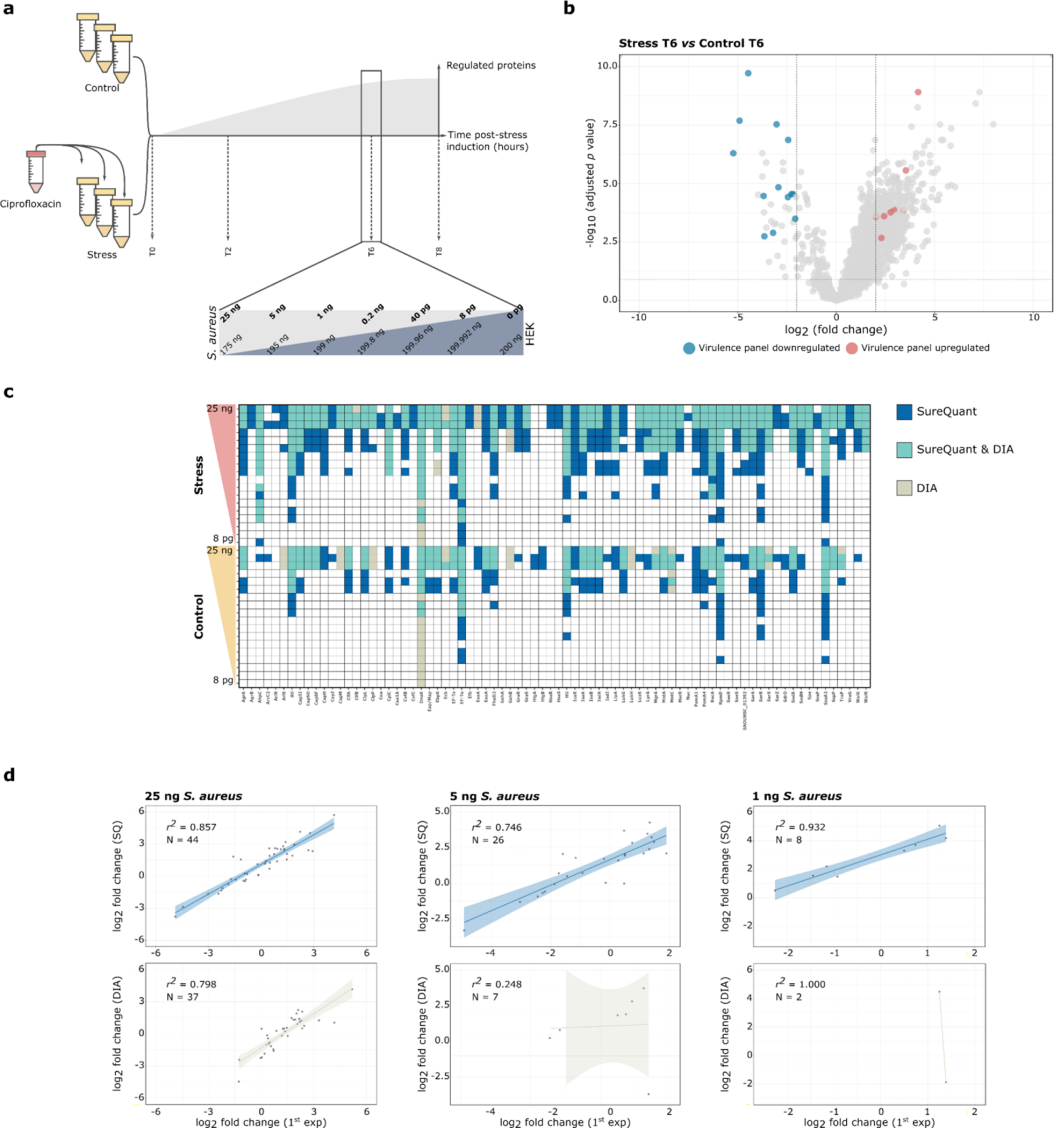
Comparative analysis of SWATH-MS and SureQuant. a) Time-points for sample harvesting, measured in hours (T0, T2, T6, and T8 h). The panel indicates the amount in ng of *S. aureus* (light gray) spiked into a HEK background (dark gray) for selected time-point (T6). b) Volcano plot for SWATH-MS based differential abundance proteome analysis of T6. The *x*-axis represents the log_2_ fold change (FC), and the *y*-axis represents the corresponding -log_10_ adjusted p-value. Proteins significantly up- and downregulated and included in the SureQuant virulence panel are marked in red and blue, respectively. The horizontal dashed line indicates the threshold for proteins where the -log_10_ adjusted *p*-value is less than 0.05, while vertical dashed lines delineate the threshold for proteins where −2 ≤ log_2_(FC) ≤ 2. Proteins that do not meet the threshold criteria (−2 ≤ log_2_(FC) ≤ 2 and -log_10_ adjusted *p*-value <0.05), or are not part of the targeted peptide set, are marked in gray. c) Heatmap for T6 illustrating the qualitative assessment of proteins quantified with SureQuant, SWATH-MS, or both. The upper panel represents stress conditions, while the lower panel represents control conditions. The amount of spiked-in *S. aureus* increased from bottom to top. d) Correlation between log_2_ fold changes of SWATH-MS in the original experiment without HEK background (*x*-axis) and the experiment with decreasing amounts of *S. aureus* in HEK background for SureQuant (blue upper panel, *y*-axis) and SWATH-MS (crude lower, *y*-axis). The number of proteins used for linear regression (*N* = ) and the R^2^ value are displayed in the upper left corner of each plot, with the amount of *S. aureus* spiked into the HEK background presented above the graph.

SureQuant has positioned itself as a versatile method that bridges the gap between standard PRM and SWATH-MS, offering a balance of comprehensiveness and sensitivity. We aimed to compare the performances of SureQuant and SWATH-MS within a biologically relevant setting. For this purpose, we cultured *S. aureus* MSSA in MHB and exposed it to ciprofloxacin, a fluoroquinolone antibiotic known to trigger the SOS response and induce expression of chromosomal virulence genes.^27,28^ We conducted a time-course experiment and collected samples at the following four time points: immediately upon ciprofloxacin addition (T0) and at 2-, 6-, and 8-h postinduction (T2, T6, T8) (Figure 4a). An initial global proteomics SWATH-MS analysis of these samples identified most protein regulations occurring at T6 and T8 (Figure S-6 and S-7). Due to potential nutritional depletion influencing results at T8, T6 was chosen for further comparative analysis between SWATH-MS and SureQuant.

To ensure the inclusion of significantly regulated proteins in our analysis, we utilized the MSstats package^29^ to perform a differential abundance analysis of stress versus control conditions, revealing 30 significantly regulated proteins. This included RecA, a crucial factor for the SOS response^30,31^ (Figure 4b and Table S-7). To compare the quantitative sensitivity of both methods, we prepared and analyzed dilutions of *S. aureus* protein extracts from T6 in a HEK background, maintaining a consistent 200 ng total protein on column while varying the *S. aureus* protein amount from 25 ng to 8 pg mimicking protein ratios in cell culture infections and infected human tissues^5,6^ (Figure 4a, Tables S-8 and S-9).

First, we carried out a qualitative comparison considering a protein identified if detected in at least two of three biological replicates. As shown in Figure 4c, SureQuant consistently identified more proteins than SWATH-MS, in particular at lower concentrations. For instance, in the 200 pg spiked stress condition sample, 8 proteins were only identified with SureQuant and not detected by SWATH-MS. Similarly, 19 out of 51 proteins were exclusively identified by SureQuant compared to only 2 by SWATH-MS when using a high concentration of 25 ng *S. aureus* proteins. Interestingly, under control conditions at 8 pg the chaperone protein DnaK was only detected by SWATH-MS, possibly due to its high abundance and the presence of additional tryptic peptides not covered by our target peptide panel.

Next, we conducted quantitative assessment of the two methods. Therefore, we correlated the fold changes determined by SureQuant and SWATH-MS with those obtained from the initial analysis of pure ciprofloxacin treated *S. aureus* samples as our reference (Table S-10 and S-11, respectively). We observed excellent ratio correlations across all concentrations using SureQuant and SWATH-MS, confirming high quantitative accuracy of both methods even at low peptide concentrations (Figure 4d). However, as already shown above, SureQuant was more sensitive and quantified around four times more proteins at the lower spiked amount of 5 and 1 ng compared to SWATH-MS. Even in 25 ng samples, only SureQuant but not SWATH-MS quantified strongly down-regulated proteins.

In summary, our findings show that SureQuant exhibits notable advantages over SWATH-MS for identifying and quantifying low abundant proteins. However, SureQuant has limitations in the number of targets that can be measured (max. 400; Figure 3c), although different panels of targets could be measured in multiple runs. By contrast, SWATH-MS can quantify thousands of peptides in a single run. The two methods thus offer complementary advantages and disadvantages.

## Conclusions

Our findings indicate that the SureQuant trigger-based acquisition method is well suited for confident identification and precise quantification of low-abundance proteins. We evaluated and optimized the most critical MS parameters of this targeted MS technique regarding specificity, sensitivity and quantitative performance. SureQuant was even more sensitive than standard SID-PRM analysis, because it allocated MS scan time more efficiently and allowed to employ more sensitive and time-consuming MS settings at similar MS cycle times. Furthermore, we determined the maximal number of targets that can be precisely quantified at different MS resolution settings with around 1000 for the fastest scan speeds representing the absolute limit of the Orbitrap LC-MS platform used. Notably, working with high scan speeds comes with the cost of MS sensitivity.^32^ Thus, for large target peptide panels, the sensitivity of SureQuant is strongly reduced and in the same range as current SWATH-MS methods. Consequently, SWATH-MS, due to its proteome wide analysis and simpler method setup, might be a more straightforward alternative if very high sensitivity is not required. This is further enhanced by the recent developments of faster scanning MS instruments that allow to apply very small SWATH-MS mass windows, which reduce peak interferences of coeluting peptides, improve quantification performance and achieve impressive proteome coverages.^33−35^ However, applying tailor-made MS settings for selected peptide quantification, as possible with SureQuant, will always provide superior analytical performance with respect to sensitivity and quantification of peptides with low abundance such as patient biopsies containing only trace amounts of *staphylococcal* or other bacterial proteins.

## Data Availability

All raw and Supporting Information are deposited in MassIVE, with Project accession number: MSV000095213

## Supporting Information Available

Details on peptide selection for SureQuant analysis, MS parameters, LC-MS/MS and SWATH-MS acquisition, PRM data acquisition and analysis, bacterial growth and sample preparation, and R code for data normalization and correction; figures on the impact of reference peptide amounts, scan distribution, calibration curves, heatmaps of protein expressions, and PCA of SWATH-MS data in the time-course experiment (PDF)
Synthetic isotope-labeled peptides and transitions, extended peptide selection and intensity thresholds, settings for SureQuant and PRM experiments, calibration curve data for PRM and SureQuant, cycle time data for various resolutions, MSstats output for SWATH analysis, comparisons of log2-fold changes between SureQuant and SWATH-MS (XLSX)
