# Supplementary material for: Optimizing SureQuant for Targeted Peptide Quantification: a Technical Comparison with PRM and SWATH-MS Methods
Source: Anal Chem. 2024 Oct 28;96(45):18061–9. doi: 10.1021/acs.analchem.4c03622 (PMC11561883; doi:10.1021/acs.analchem.4c03622)
Supplement: Supplementary file 1 — ac4c03622_si_001.pdf [file ac4c03622_si_001.pdf]

Supporting Information to

Optimizing SureQuant for Targeted Peptide Quantification: A Technical Comparison with PRM and SWATH-MS Methods

Minia Antelo-Varela*, Dirk Bumann, Alexander Schmidt*

Biozentrum, University of Basel, Spitalstrasse 41 CH-4056, Basel, Switzerland.

Correspondence and requests for materials should be addressed to A.S. (Phone: +41 61 207 20 59, email: alex.schmidt@unibas.ch) or M.A.V. (Phone: +41 61 207 71 38, email:, minia.antelovarela@unibas.ch).

Table of Contents

DESCRIPTION OF SUPPORTING INFORMATION S2

SUPPORTING MATERIAL & METHODS S3

Selection of peptides for SureQuant analysis S3

Survey MS parameters S3

Survey MS analysis S3

LC/MS-MS analysis S4

PRM data acquisition S4

SWATH-MS Acquisition of *S. aureus* S4

Extraction of number of scans S4

PRM data analysis S4

Determination of optimal triggering signal S5

Optimizing Intensity Threshold Settings for SureQuant Analysis S5

SWATH-MS data analysis S5

Bacterial growth & sample preparation S5

Rcode for data normalization and correction S6

SUPPORTING FIGURES S9

Figure S1 ..... S9

Figure S2. ..... S10

Figure S3. ..... S11

Figure S4. ..... S12

Figure S5. ..... S13

Figure S6. ..... S14

Figure S7. ..... S15

SUPPORTING REFERENCES S16

DESCRIPTION OF SUPPORTING INFORMATION

This Supporting Information contains additional information regarding selection of peptides for SureQuant analysis, survey MS parameters and analysis, LC-MS/MS, PRM data acquisition, SWATH-MS acquisition of *S. aureus*, extraction of number of scans, PRM data analysis, determination of optimal triggering signal, optimization of intensity threshold settings for SureQuant analysis, SWATH-MS analysis, bacterial growth & sample preparation and the Rcode used for normalization and correction of SureQuant data. It includes seven figures illustrating the impact of reference peptide amount on identification and quantification (Figure S1), distribution of scans based on mass accuracy settings for samples and blanks and impact on quantification accuracy (Figure S2), histogram depicting the $\log_{10}$ MS1 peak heights of reference peptides (Figure S3), distribution of scans by intensity threshold settings and their effect on quantification accuracy (Figure S4), calibration curve for IsdB, SasA and IcaR (Figure S5), heatmap of protein expressions identified and quantified via SWATH-MS in the time-course experiment (Figure S6) and principal component analysis (PCA) of SWATH-MS in the time-course proteomic analysis of *S. aureus* in control and stress conditions (Figure S7). In addition, the Supporting Information also includes eleven excel tables with the following data: list of Synthetic isotope–labeled peptides and respective transitions (Table S1.1 & S1.2), list of extended peptide selection including precursor m/z and intensity threshold (Table S2), list of selected SIL peptides with corresponding intensity thresholds (Table S3), list of settings used for SureQuant and PRM experiments (Table S4.1 and S4.2, respectively), coefficient of variation (CV) and average percent bias (PBav) for both PRM and SureQuant, calculated using the CalibraCurve script for the selected peptides (Table S5), list of cycle time in ms consumed for each target at different resolutions (Table S6), MSstats output of SWATH analysis of time-course experiment (Table S7), MSstats output of SWATH analysis of time-point 6 for different amounts of *S. aureus* injected on column (Table S8), copy/cell of targeted proteins acquired with SureQuant for different amounts of *S. aureus* injected on column (Table S9), comparison of $\log_2$ fold-change between SureQuant and SWATH-MS from the initial analysis of pure ciprofloxacin treated *S. aureus* samples (Table S10) and comparison of $\log_2$ fold-change between SWATH-MS and SWATH-MS from the initial analysis of pure ciprofloxacin treated *S. aureus* samples (Table S11).

SUPPORTING MATERIAL & METHODS

Selection of peptides for SureQuant analysis

We included a total of 131 proteins comprising major virulence factors, vaccine targets, resistance markers and metabolic proteins of the human pathogen *S. aureus*. The list of labeled peptides and respective transitions are detailed in the Supporting Information Table S1.1 and 1.2. Peptides were selected based on SWATH-MS data previously generated in the lab (data now shown). Peptides that were identified with high confidence and that met a series of criteria (no methionine, lysine (K) or arginine (R) not before proline (P), length be-tween 7 and 21 amino acid residues, tryptic peptides, no missed cleavages) were chosen for following experiments. When no experimental data was available for these proteins, an in silico digest was performed and 5 to 8 candidate pep-tides/protein that followed the criteria described above, were selected. This comprised a total of 326 S. aureus peptides. Synthetic isotope–labeled C-terminal K or R SIL peptides were purchased from JPT Peptide Technologies (Berlin, Germany), using the SPOT synthesis technology[1]. The unlabeled version of these peptides (UL) were obtained with identical specifications and purchased from the same company. The pool of synthesized peptides was dissolved in 0.1% trifluoroacetic acid (TFA) in 20% (v/v) acetonitrile/water to a final concentration of 1 pmol/µL, and stored at -20°C. A working solution was prepared and dissolved in 0.1% (v/v) formic acid/water to a final concentration of 50 fmol/ µL and subjected to nano-LC/MS-MS analysis to determine the MS1 intensity of 326 peptides.

Survey MS parameters

Prior to conducting SureQuant acquisition, the heavy-labeled peptides underwent initial characterization through DDA. A mixture containing 400 fmol of each heavy-labeled peptide was injected for this purpose. The mass spectrometry parameters employed for these preliminary analyses were as follows: spray voltage set to 2.5 kV, with no sheath or auxiliary gas flow, and a heated capillary temperature of 275°C. DDA analysis involved collecting full-scan mass spectra within an m/z range of 350-1600, with an AGC target value of 300%, maximum injection time (IT) of 25 ms, and a resolution of 120,000. For each scan, the top 38 most intense ions on the inclusion list (if above a 5e3 intensity threshold) were isolated using an isolation width of 1.4 m/z and fragmented by higher-energy collisional dissociation (HCD) with a normalized collision energy (nCE) of 27%. Fragmentation parameters included a maximum IT of 25 ms, AGC target value set to standard, and a resolution of 15,000.

Survey MS analysis

Before conducting SureQuant acquisition, the heavy-labelled peptides underwent initial characterization using DDA. This involved assessing precursor charge states ranging from +2 to +5 for each heavy-labelled trigger peptide to determine the most suitable charge states and product ions for subsequent targeted experiments Supporting Information (Table S1.1 and S1.2). For data processing, raw data were imported into MaxQuant (1.6.23)[2] incorporated with an Andromeda search engine[3]. Database searches were carried out against a *S. aureus* database (UP000008816) with common contaminants and iRT added by MaxQuant. The database search was performed with the following parameters: peptide tolerance, 20 ppm; primary digest reagent, trypsin; missed cleavages, 2; fixed modification, carbamidomethyl C (+57.0215); variable modifications, oxidation M (+15.9949), heavy labeled R (+10.00827), heavy labeled K (+8.0142). Results were filtered for a 50% false discovery rate (FDR) on spectrum level and 100% FDR on protein level. Subsequently, an in-house script was employed to extract transition details (precursor m/z and fragment peptides) for the selected peptides. This information was then integrated into the method file of the SureQuant acquisition template, available in the Thermo Orbitrap Exploris Series 4.1 instrumentation.

LC/MS-MS analysis

Samples were separated on a Dionex UltiMate 3000 system (ThermoFisher Scientific) coupled online to an Orbitrap Exploris 480 mass spectrometer (ThermoFisher Scientific). In-house packed 20 cm, 75 μm ID capillary column with 1.9 μm Reprosil-Pur C18 beads (Dr. Maisch, Ammerbuch, Germany) was used. The column temperature was maintained at 50 °C using an integrated column oven interfaced online with the mass spectrometer. Formic acid (FA) 0.1% was used to buffer the pH in the two running buffers used. The total gradient time was 60 min and went from 2% to 12% acetonitrile (ACN) in 5 min, followed by 45 min to 35%, and 10 min at 50%. This was followed by a washout by 95% ACN, which was kept for 20 min, followed by re-equilibration of 0.1% FA buffer. Flow rate was kept at 300 nL/min.

PRM data acquisition

24 pre-selected SIL peptides were analyzed by scheduled-PRM Each sample was analyzed in triplicate in order of increasing concentration, targeting a selected set of 24 peptides in scheduled PRM mode (see Table S4.2 for collision energies and scheduling). Samples were measured on the Exploris 480 MS coupled to UltiMate 3000 RSLC Nano LC system as previously described, using the SureQuant gradient. Standard MS parameters were as follows: 2.5 kV; no sheath or auxiliary gas flow; heated capillary temperature, 275 °C. Samples were acquired with MS1 resolution of 120,000, scan range 375-1600, AGC target value: 300% (3e6). Targeted peptides were isolated [isolation width 0.4 m/z] and fragmented [nCE 30%] by HCD with Orbitrap resolution set to 15,000, 22 ms maximum IT for the heavy peptide, and 60,000, 116 ms maximum IT for the light peptide.

SWATH-MS Acquisition of *S. aureus*

Samples were separated on a Dionex UltiMate 3000 system (ThermoFisher Scientific) coupled online to an Orbitrap Exploris 480 mass spectrometer (ThermoFisher Scientific). In-house packed 20 cm, 75 μm ID capillary column with 1.9 μm Reprosil-Pur C18 beads (Dr. Maisch, Ammerbuch, Germany) was used. The column temperature was maintained at 60 °C using an integrated column oven interfaced online with the mass spectrometer. Formic acid (FA) 0.1% was used to buffer the pH in the two running buffers used. The total gradient time was 60 min and went from 2% to 12% acetonitrile (ACN) in 5 min, followed by 45 min to 35%, and 10 min at 50%. This was followed by a washout by 95% ACN, which was kept for 20 min, followed by re-equilibration of 0.1% FA buffer. Flow rate was kept at 300 nL/min. Spray voltage was set to 2500 V, funnel RF level at 40, and heated capillary at 275 °C. For DIA experiments full MS resolutions were set to 120,000 and full MS normalized AGC target was 300% with an IT of 45 ms. Mass range was set to 350–1400. Normalized AGC target value for fragment spectra was set at 1000%. In all, 63 windows of 9 Da were used with an overlap of 1 Da. Resolution was set to 15,000 and IT to 22 ms. Normalized CE was set at 28%. All data were acquired in centroid mode using positive polarity and advanced peak determination was set to on.

Extraction of number of scans

To determine the total number of MS1 and MS2 scans, we generated an additional raw file for each condition comprising only the relevant measurement time (excluding equilibration) using FreeStyle (version 1.7). Subsequently, this file was imported into RawMeat (version 2.1) to extract the number of scans.

PRM data analysis

Raw data was imported into SpectroDive (10.4.210316.47784 (Ictíneo II) and searched against the pre-selected set of peptides. Endogenous peptides were considered for further quantification if the elution group *q*-value < 0.01. The analyses were conducted using Rstudio version 4.3.2. In our comparison between PRM and SureQuant, we employed CalibraCurve[4], a tool facilitating automated batch-mode assessment of dynamic linear ranges and quantification limits. Utilizing the standard settings integrated into the Rscript, we set the minimum number of replicates to two, with CV threshold and the maximum

allowed percent bias threshold both configured at 20%. For determining central tendency, the mean was utilized. Calculation method for the preliminary linear range was set as TRUE, indicating all CV values within the preliminary range must pass the CV threshold criteria.

Determination of optimal triggering signal

Determining the optimal triggering signal involves finding a balance between the minimal amount of reference peptide required to trigger the instrument and the maximum amount that can be injected without compromising the assay's sensitivity. In this study, 24 peptides were pre-selected, and decreasing amounts of reference SIL peptides were spiked in across five orders of magnitude (0.04, 0.2, 1, 5, and 10 fmol), while maintaining a constant amount of endogenous peptide (10 fmol) in a 250 ng HEK (Human embryonic kidney cell line) background. Analysis was performed using an Exploris 480 MS coupled with an UltiMate 3000 RSLC Nano LC system, following the SureQuant gradient method. The standard MS parameters for SureQuant acquisition were set as follows: a spray voltage of 2500 V, no sheath or auxiliary gas flow, and a heated capillary maintained at 275 °C. Full-scan mass spectra were acquired with a scan range of 375-1600 m/z, an AGC target value of 300%, maximum injection time (IT) of 50 ms, and a resolution of 120,000. Within a 5-second cycle time per MS1 scan, heavy peptides matching the m/z (within 10 ppm) were isolated (isolation width of 0.4 m/z) and subjected to fragmentation (nCE: 27%) by HCD with a scan range of 150-1700 m/z, maximum IT of 54 ms, AGC target value of 1000%, and a resolution of 30,000. A product ion trigger filter was then applied, conducting pseudo-spectral matching and triggering MS/MS events exclusively for the endogenous target peptide at the defined mass offset if at least two product ions were detected from the specified list. If triggered, the subsequent MS/MS scan for the light peptide shared the same collision energy (CE), scan range, and AGC target as the heavy trigger peptide, with an increased maximum injection time and resolution (IT: 256 ms, resolution: 120,000).

Optimizing Intensity Threshold Settings for SureQuant Analysis

During SureQuant analyses, a high-resolution MS1 scan is conducted to monitor the predefined optimal precursor ions of the SIL peptides, utilizing a list of associated m/z values and intensity thresholds. To determine the optimal intensity threshold setting, we introduced an equal amount of SIL and endogenous peptides (10 fmol/ul each) into a background of 250 ng/ul of HEK. The analysis was carried out with the same parameters outlined in the "SureQuant Acquisition" section, but this time, intensity threshold filters were incorporated. Initially, we evaluated different stringencies: no intensity threshold, 1e5, 1e6, 1e7, and 1e8. Furthermore, we utilized the MS1 monoisotopic peak height derived from experimental data obtained with optimized injection amounts of SIL peptides (10 fmol) to calculate the median monoisotopic peak height. Subsequently, this value was employed to generate various panels with specific intensity thresholds for each peptide, as well as thresholds set at 5 times and 25 times less than the previously determined monoisotopic peak height (Table S4).

SWATH-MS data analysis

For data processing and protein identification, raw data were imported into SpectroNaut (16.1.220730.53000, Biognosys) and analyzed with directDIA. Searches were carried out against a fasta file including the proteomes of *S. aureus* (UP000008816) and Human (UP000005640), when relevant. Cysteine carbamidomethylation was set as fixed modification. Methionine oxidation, methionine excision at the N terminus were selected as variable modifications. Method evaluation was selected. Results were filtered for a 1% false discovery rate (FDR) on spectrum, peptide, and protein levels. Relative protein abundances were calculated using the MSstats package[5] with default settings selected.

Bacterial growth & sample preparation

Staphylococcus aureus cells were grown in Mueller Hinton Broth (MHB) medium. Exponentially growing cells (optical density at 600 nm [OD_{600}] of 0.5) were challenged with 5 µg/mL ciprofloxacin (v/v), and samples were harvested at 0, 2, 6, 8 hours after treatment. Control cells, to which no

ciprofloxacin was added, were collected at the same time point. Three biological replicates were processed for each time-point. Cells were harvested by centrifugation (10,000 x *g* for 5 min at 4 °C), and cell pellets were washed once with Phosphate-buffered saline (PBS, Life Technologies). Cell pellets were ressuspended in lysis buffer (10% Sodium dodecyl sulfate (SDS), 20mM tris(2-carboxyethyl) phosphine (TCEP), 200 mM Triethyloammonium bicarbonate (TEAB)) followed by incubation at 95°C for 10 min. Cells were disrupted by ultrasonication using the PIXUL system (Active Motif) for 30 min with default settings (pulse 50 cycles, PRF 1 kHz, burst rate 20 Hz)) and the protein content was determined by tryptophan-based fluorescence assay (Infinite M Plex, Tecan). Sample alkylation was performed by addition of 20 mM iodoacetamide (IAA) and incubation at 25°C for 30 min with gentle shaking. 20 µg of material was used for protein digestion using the S-Trap protocol according to the manufacturer (ProtiFi). Peptide concentration was determined using an UV-based assay (Infinite M Nano, Tecan). Prior to analysis, SIL were added to the samples to a final concentration of 4 fmol/µL. In addition, we have prepared an aliquot containing only SIL peptides, which were used as a blank to calculate possible contamination on the light channel.

Rcode for data normalization and correction

```
# ---- ID ---------------------------

# Script developed by Minia Antelo
# SpectroDive output file
# Analysis of SureQuant data for P515 samples

# ---- Load library ------------------

df01   <-   read.csv("file   location/filename.csv",   header=TRUE,
stringsAsFactors=FALSE) # SD report

# ---- Define functions ---------------

geomean <- function(x, na.rm = FALSE, trim = 0, ...) {
  exp(mean(log(x, ...), na.rm = na.rm, trim = trim, ...))
}

geosd <- function(x, na.rm = FALSE, ...) {
  exp(sd(log(x, ...), na.rm = na.rm, ...))
}

# ---- Filter blank samples -----------

# Filter signal from SIL peptides measured in the experiment
blank <- df01 %>% filter(R.Condition == "0ng_SA")
blank.L <- filter(blank, TG.Reference %in% "False")
blank.L  <-  blank.L  %>%  rename(JPT_Ratio  =  EG.TargetReferenceRatio,
JPT_Quantity = TG.RawQuantity)

# ---- Calculate geometric mean of SIL contamination ----

Geo_blank <- blank.L %>%
  group_by(EG.StrippedSequence) %>%
  summarise(
    n=n(),
    mean=mean(JPT_Ratio),
    geom=exp(mean(log(JPT_Ratio))),
    sd=sd(JPT_Ratio)
  ) %>%
  mutate( se=sd/sqrt(n), ic=se * qt((1-0.05)/2 + .5, n-1))
```

```
Geo_blank_R <- Geo_blank %>% select(EG.StrippedSequence, geom, sd)

# ---- Filter data for light peptides -----------

df01L <- df01 %>% filter(TG.Reference == "False")
df01L.1 <- df01L[!grepl("0ng_SA", df01L$R.Condition),]

# ---- Calculate number of IDs using Elution Group q-value ---------

df01L.1 <- df01L.1 %>% mutate(IDs = ifelse(EG.Qvalue > 0.01, 0, 1))

# ---- Integrate SIL contamination data ---------

df01L.2     <-     inner_join(df01L.1,     Geo_blank_R,     by     =
"EG.StrippedSequence")
df01L.2$Sample <- paste(df01L.2$R.Condition, df01L.2$R.Replicate)

# ---- Calculate endogenous vs SIL peptide ratios --------

df01L.2$EndVsJPT <- df01L.2$EG.TargetReferenceRatio / df01L.2$geom

# ---- Identify peptides using both EG q-value and Endogenous vs SIL
Ratio --------

df01L.2 <- df01L.2 %>% mutate(Ratio = ifelse(EndVsJPT > 2, 1, 0))
df01L.2$Criteria <- df01L.2$IDs + df01L.2$Ratio
df01L.3 <- df01L.2 %>% filter(Criteria == 2)

# ---- Data normalization -----------

# Subtracting the determined contamination H/L ratios of blank samples
df01L.3 <- df01L.3 %>% mutate(minusCont = EG.TargetReferenceRatio -
geom)

# ---- Median normalization -----------

df01L.MED <- df01L.3 %>%
  group_by(R.Condition) %>%
  summarise(Median = median(minusCont),
            SD = sd(minusCont),
            CV = (SD/Median)*100) %>%
  select(R.Condition, Median)

df01L.4 <- inner_join(df01L.3, df01L.MED, by = 'R.Condition')
df01L.4$MedianNorm <- df01L.4$minusCont / df01L.4$Median

# ---- Data correction using Avogadro's constant ----

Avogadro <- 6.0221408E+23
fmoltomol <- 1E-15

df01L.4 <- df01L.4 %>% mutate(fmoloncolumn = MedianNorm * 10)

df01L.5 <- df01L.4 %>%
  group_by(Sample, PG.ProteinId) %>%
  arrange(desc(fmoloncolumn), .by_group = TRUE)
```

```
df01L.5 <- df01L.5 %>% group_by(Sample, PG.ProteinId) %>% top_n(1,
fmoloncolumn)

df01L.6 <- inner_join(df01L.4, df01L.5, by = c('Sample',
'PG.ProteinId'))

# ---- Adjust ratios based on fmoloncolumn ----

df01L.6 <- df01L.6 %>% mutate(RatioFactor = fmoloncolumn.x /
fmoloncolumn.y)

df01L.7 <- df01L.6 %>%
  group_by(PG.ProteinId, EG.StrippedSequence.x) %>%
  summarise(MedianFactor = median(RatioFactor))

df01L.8 <- inner_join(df01L.6, df01L.7, by = c('PG.ProteinId',
'EG.StrippedSequence.x'))
df01L.8 <- df01L.8 %>% mutate(RatioAdjusted = fmoloncolumn.x /
MedianFactor)

# ---- Calculate protein copy number ----

df01L.9 <- df01L.8 %>%
  group_by(Sample, PG.ProteinId) %>%
  summarise(GeoMeanProtein = geomean(RatioAdjusted),
            SDProtein = sd(RatioAdjusted),
            CVProtein = (SDProtein / GeoMeanProtein) * 100)

df01L.9 <- df01L.9 %>%
  mutate(Molecules = GeoMeanProtein * Avogadro * fmoltomol) %>%
  mutate(MoleculesLog = log10(Molecules))

df01L.10 <- df01L.9 %>% select(Sample, PG.ProteinId, Molecules)

# ---- Pivot table for output ----

df01L.10 <- pivot_wider(df01L.10, names_from = Sample, values_from =
Molecules)
```

SUPPORTING FIGURES

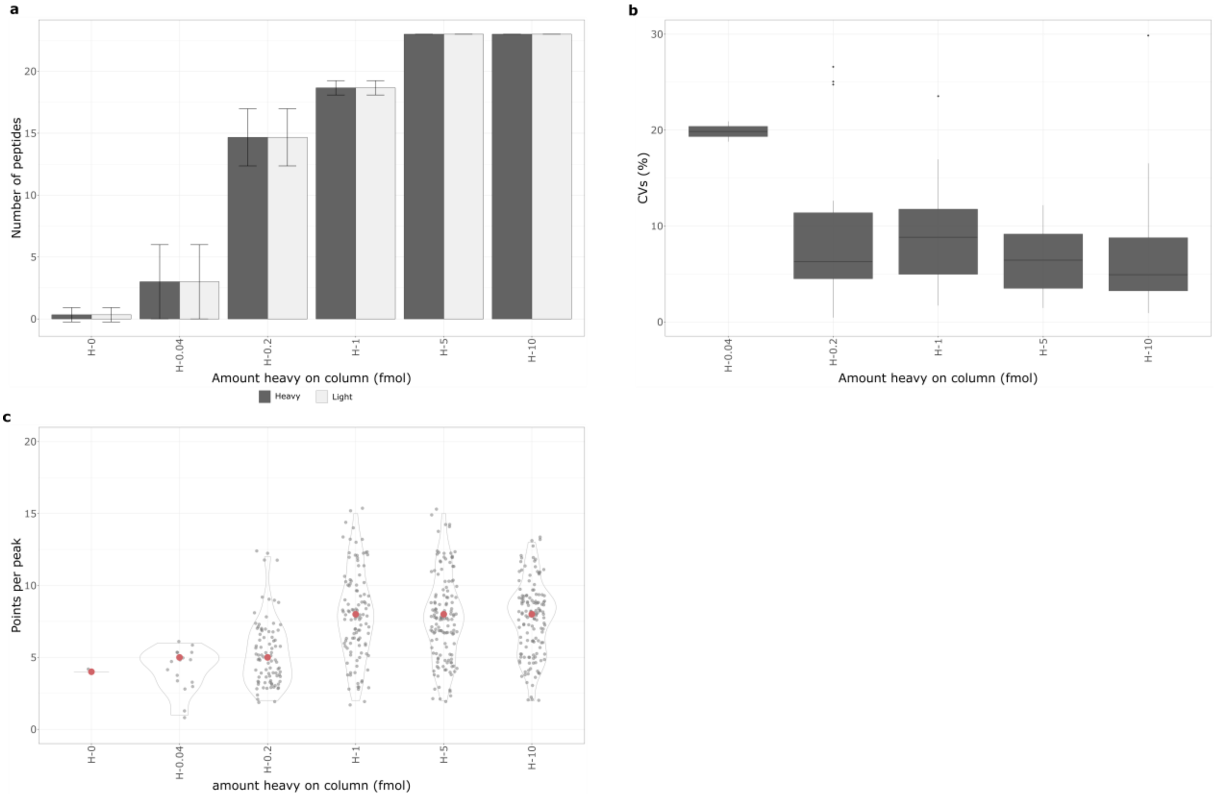

Figure S1. Impact of reference peptide amount on identification and quantification. a) The graph displays the average identifications from triplicate technical replicates in relation to the amount of reference peptide injected onto the column. Dark grey bars denote the number of heavy peptides identified, while light grey bars represent the identification of light peptides. Error bars indicate standard deviations. b) A box plot shows the CVs for all identified peptides based on the amount of heavy peptide applied to the column. c) A violin plot illustrates the distribution of data points per peak for all identified peptides, corresponding to the amount of heavy peptide used. A red circle marks the average number of points per peak for each condition.

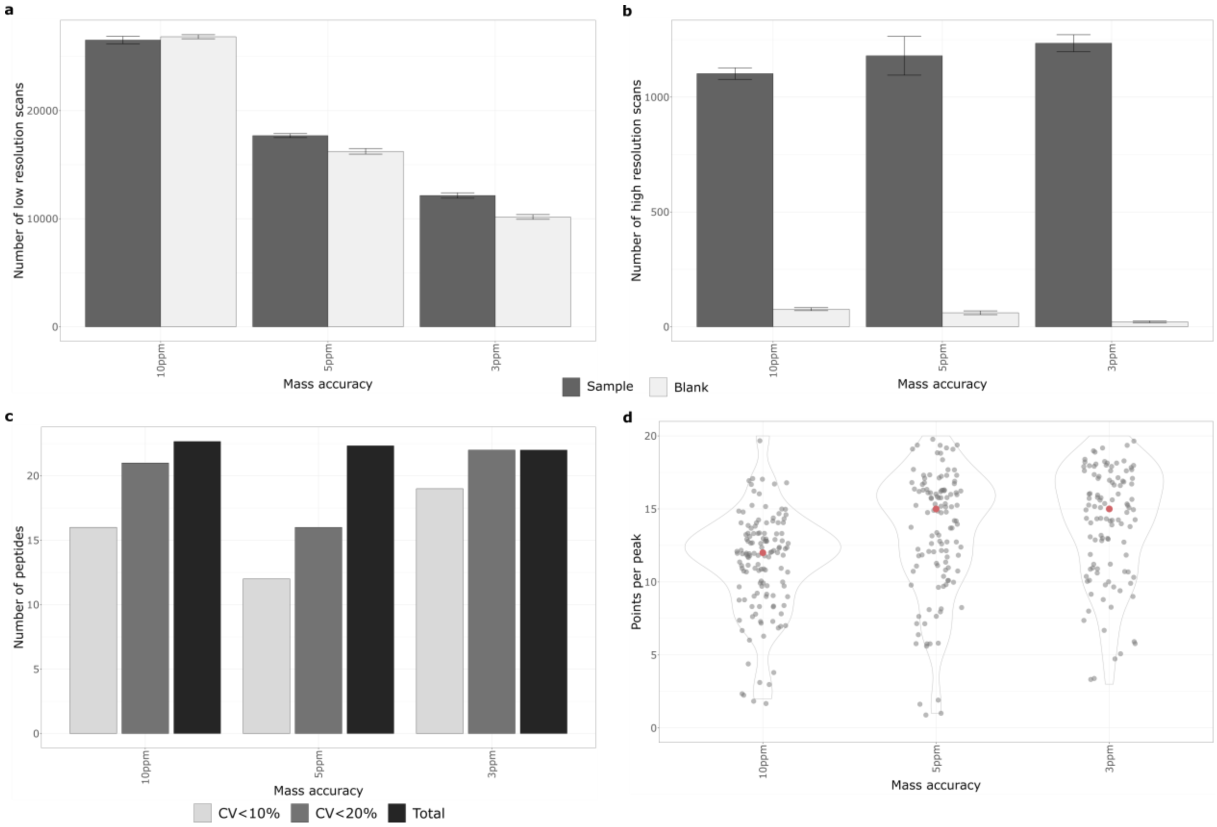

Figure S2. Distribution of scans based on mass accuracy settings for samples and blanks and impact on quantification accuracy. a) Counts of low-resolution scans. b) Counts of high-resolution scans. Results are shown with dark grey bars representing samples containing both light and heavy peptides in a 250 ng HEK background, and light grey bars representing blank samples consisting solely of the 250 ng HEK background. The error bars display the standard deviations. c) The average identifications of triplicate technical replicates and the number of peptides with CVs below defined thresholds were calculated. d) Violin plot visualization of number of points per peak of all identified peptides according to the applied mass accuracy setting.

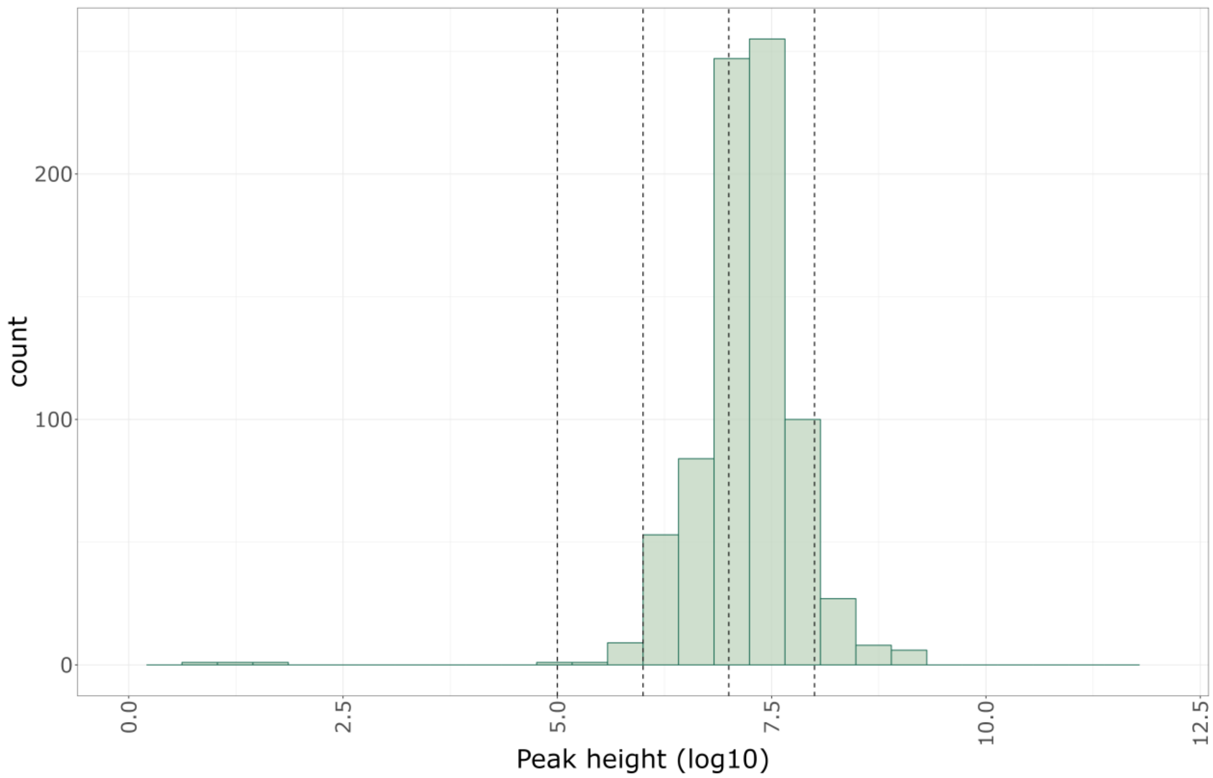

Figure S3. Histogram depicting the $\log_{10}$ MS1 peak heights of reference peptides (10 fmol on column). A dashed black line indicates the intensity threshold filters that were applied.

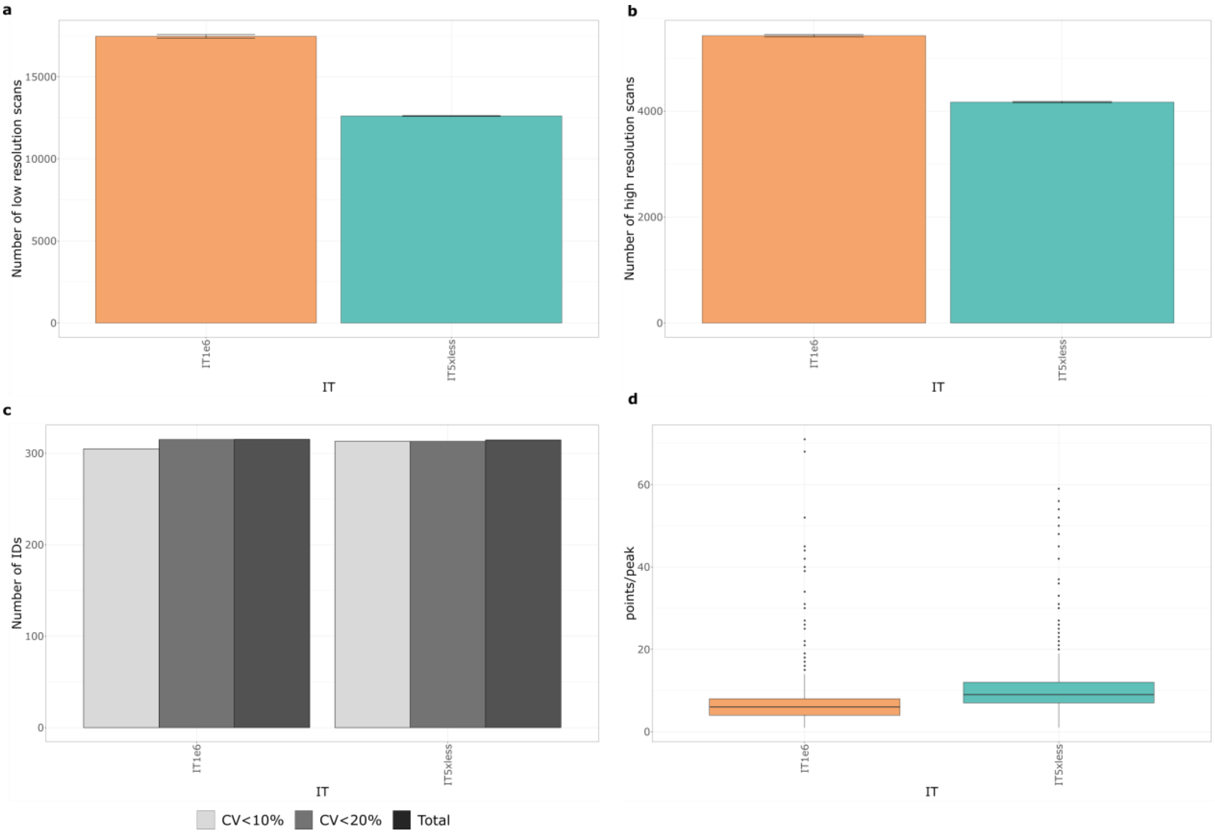

Figure S4. Distribution of scans by intensity threshold settings and their effect on quantification accuracy. a) Number of low-resolution scans. b) Number of high-resolution scans, with orange bars indicating samples using a 1e6 intensity threshold filter and green bars showing samples with an adjusted intensity threshold filter for each reference peptide. c) The average number of identifications from triplicate technical replicates and the count of peptides with CVs below specified thresholds. d) Box plot depiction of the number of data points per peak for all identified peptides, based on the intensity threshold setting applied.

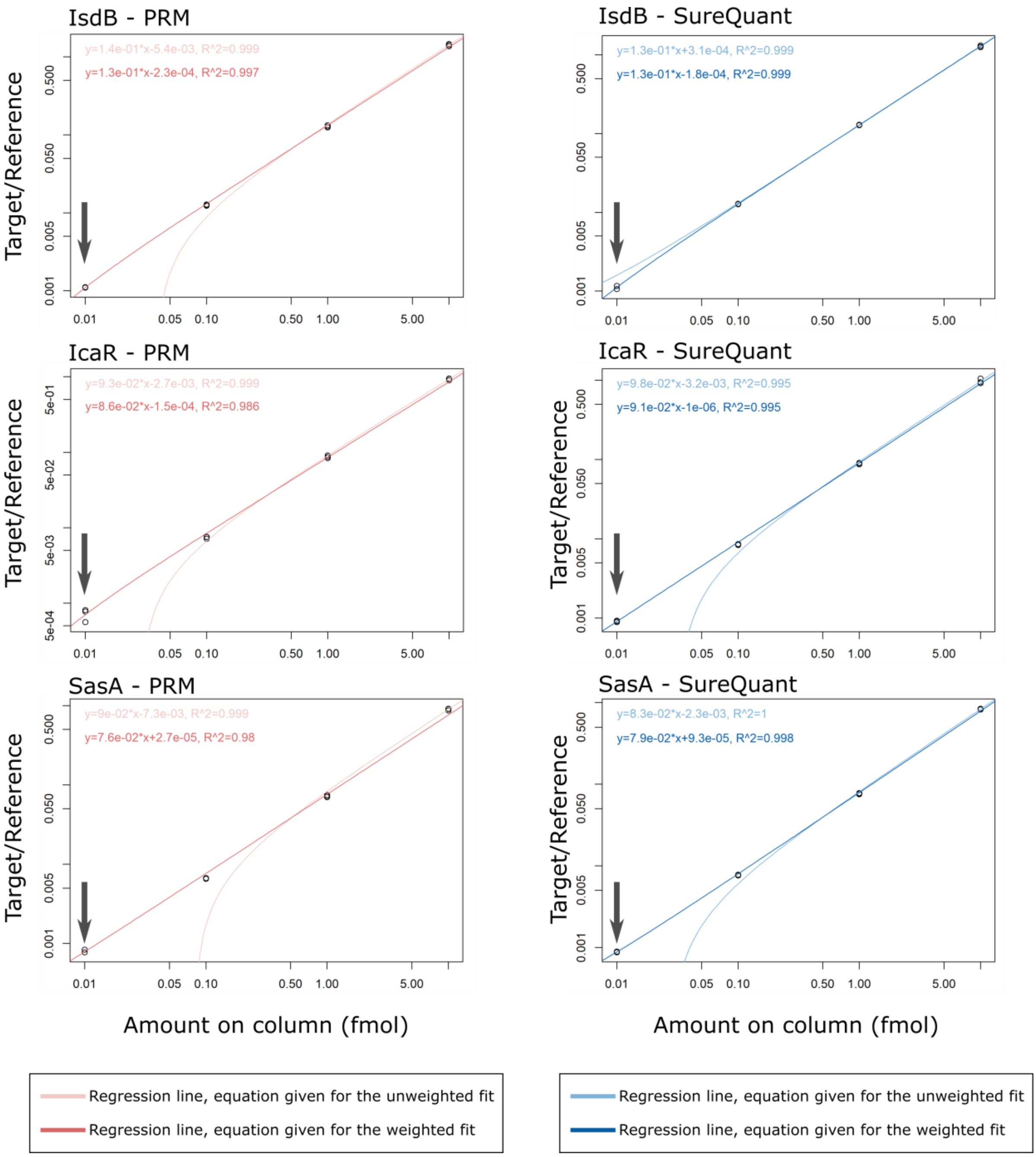

Figure S5. Calibration curve for a transition of the GEVESSSTTPTK peptide (protein IsdB), FGAVEESK peptide (protein IcaR) and YVDVTTGK (protein SasA) using CalibraCurve[4]. Red color depicts results for data acquired with PRM and blue corresponds to data acquired with SureQuant.

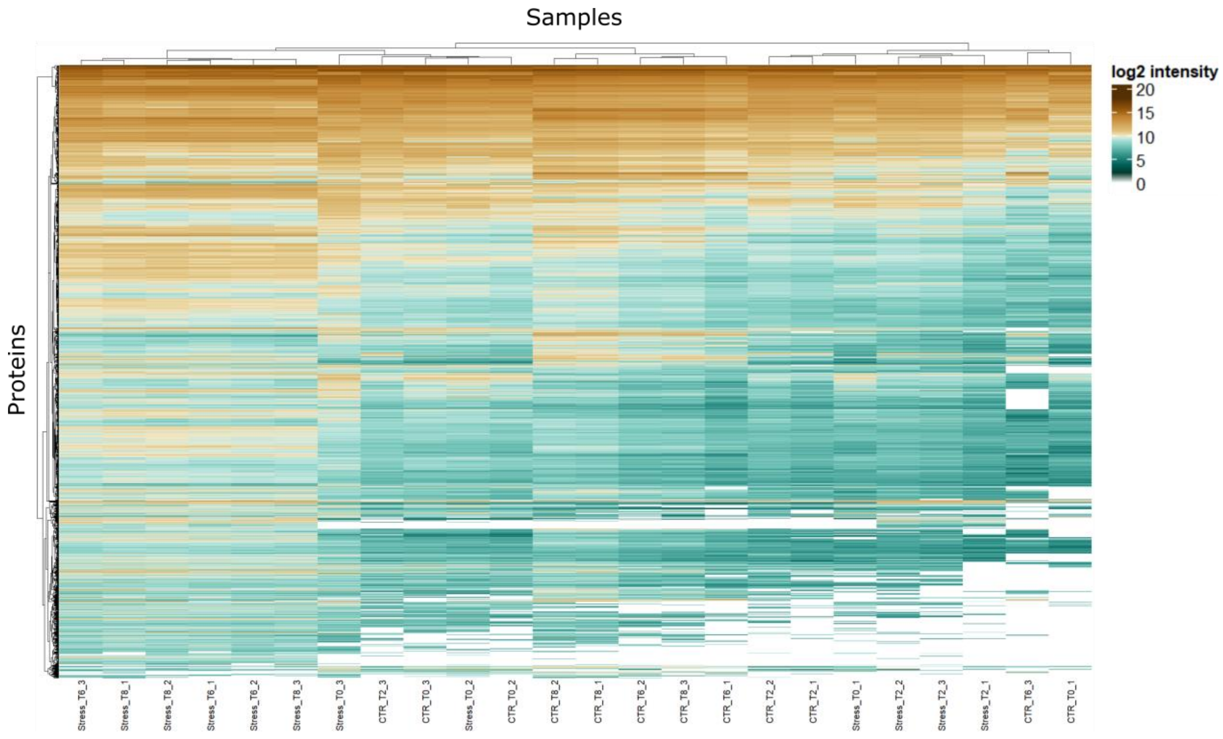

Figure S6. Heatmap of protein expressions identified and quantified via SWATH-MS in the time-course proteomic analysis of *S. aureus* under control and stress conditions (CTR and Stress, respectively). Each column in the heatmap corresponds to a sample, and each row corresponds to a specific protein (n = 1993). The color scale transitions from green to brown to indicate protein expression levels ranging from low to high (log_2 intensity).

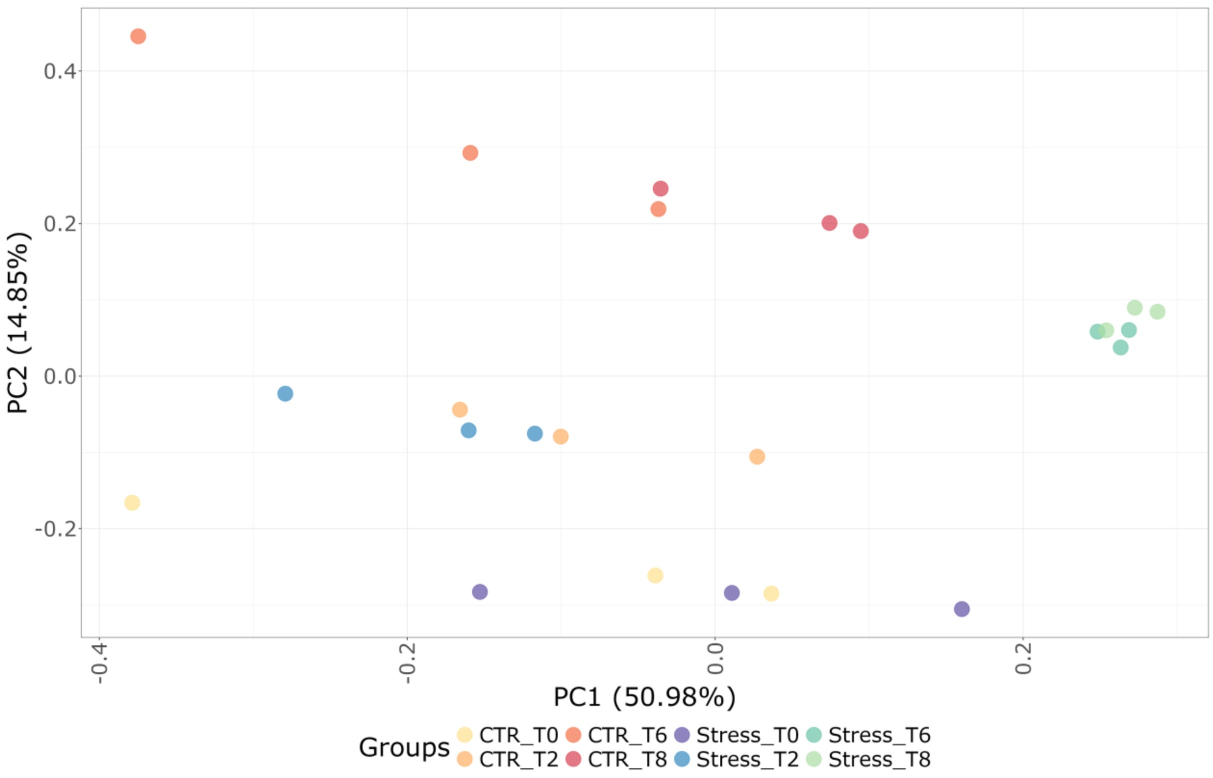

Figure S7. Principal component analysis (PCA) of SWATH-MS in the time-course proteomic analysis of *S. aureus* in control and stress conditions (CTR and Stress, respectively) at different time points encompassing a total of eight categories. Samples are colored accordingly. Results were illustrated using the factoextra R package.

SUPPORTING REFERENCES

(1) Hilpert, K.; Winkler, D. F. H.; Hancock, R. E. W. Peptide Arrays on Cellulose Support: SPOT Synthesis, a Time and Cost Efficient Method for Synthesis of Large Numbers of Peptides in a Parallel and Addressable Fashion. *Nat Protoc* **2007**, *2* (6), 1333–1349.

(2) Tyanova, S.; Temu, T.; Cox, J. The MaxQuant Computational Platform for Mass Spectrometry-Based Shotgun Proteomics. *Nature Protocols* **2016**, *11* (12), 2301–2319.

(3) Cox, J.; Neuhauser, N.; Michalski, A.; Scheltema, R. A.; Olsen, J. V.; Mann, M. Andromeda: A Peptide Search Engine Integrated into the MaxQuant Environment. *Journal of Proteome Research* **2011**, *10* (4), 1794–1805.

(4) Kohl, M.; Stepath, M.; Bracht, T.; Megger, D. A.; Sitek, B.; Marcus, K.; Eisenacher, M. CalibraCurve: A Tool for Calibration of Targeted MS-Based Measurements. *Proteomics* **2020**, *20* (11).

(5) Choi, M.; Chang, C. Y.; Clough, T.; Broudy, D.; Killeen, T.; MacLean, B.; Vitek, O. MSstats: An R Package for Statistical Analysis of Quantitative Mass Spectrometry-Based Proteomic Experiments. *Bioinformatics* **2014**, *30* (17), 2524–2526.
